# Generic medicines and generic substitution: contrasting perspectives of stakeholders in Ireland

**DOI:** 10.1186/s13104-015-1764-x

**Published:** 2015-12-15

**Authors:** A. O’Leary, C. Usher, M. Lynch, M. Hall, L. Hemeryk, S. Spillane, P. Gallagher, M. Barry

**Affiliations:** National Centre for Pharmacoeconomics, St. James’s Hospital, Dublin 8, Ireland; School of Pharmacy, Royal College of Surgeons, St. Stephens Green, Dublin 2, Ireland; Department of Pharmacology and Therapeutics, St. James’s Hospital, Dublin 8, Ireland

**Keywords:** Generic medicines, Generic substitution, Knowledge, Perception, Key stakeholders

## Abstract

**Background:**

The Health (Pricing and Supply of Medical Goods) Act 2013 passed into law in July 2013 and legislated for generic substitution in Ireland. The aim of the study was to ascertain the knowledge and perceptions of stakeholders i.e. patients, pharmacists and prescribers, of generic medicines and to generic substitution with the passing of legislation.

**Methods:**

Three stakeholder specific questionnaires were developed to assess knowledge of and perceptions to generic medicines and generic substitution. Purposive samples of patients, prescribers and pharmacists were analysed. Descriptive quantitative and qualitative analyses were undertaken.

**Results and discussion:**

A total of 762 healthcare professionals and 353 patients were recruited. The study highlighted that over 84 % of patients were familiar with generic medicines and are supportive of the concept of generic substitution. Approximately 74 % of prescribers and 84 % of pharmacists were supportive of generic substitution in most cases. The main areas of concern highlighted by the healthcare professionals that might impact on the successful implementation of the policy, were the issue of bioequivalence with generic medicines, the computer software systems used at present in general practitioner (GP) surgeries and the availability of branded generics. The findings from this study identify a high baseline rate of acceptance to generic medicines and generic substitution among patients, prescribers and pharmacists in the Irish setting. The concerns of the main stakeholders provide a valuable insight into the potential difficulties that may arise in its implementation, and the need for on-going reassurance and proactive dissemination of the impact of the generic substitution policy.

**Conclusion:**

The existing positive attitude to generic medicines and generic substitution among key stakeholders in Ireland to generic substitution, combined with appropriate support and collaboration should result in the desired increase in rates of prescribing, dispensing and use of generic medicines.

## Background

The escalating cost of public provision of prescription medicines has become a global challenge [1, 2]. In Ireland, prescription medicines are provided to citizens free of charge or on a subsidised basis through the taxation-based public health service. State expenditure on prescribed medicines has grown steadily since the introduction of the Health Act, 1970, which provided for the provision of prescribed medicines free of charge to eligible persons. This act resulted in the establishment of the General Medical Services (GMS) scheme, which covers approximately 40 % of the Irish population as deemed eligible by means testing [3]. For persons not covered under the GMS scheme, State subsidisation of medicine costs occurs through the ‘community drug schemes’, which include the Drugs Payment (DP) scheme, Long Term Illness (LTI) scheme and High Tech Drug (HTD) scheme. The State pays the cost of regular medicines above and beyond a threshold per family which stands at €144 per month as of 2015 (DP scheme). The State also bears the cost of complex expensive drugs such as oncology medicines dispensed in the community (HTD). Additionally, the State pays the cost of drugs prescribed for specified medical conditions which are included within the LTI scheme, for example, diabetes mellitus or epilepsy. Medicines are provided to patients through the above schemes by community pharmacies which enter into contractual arrangements with the Irish health service. Drug costs borne by the pharmacies are recouped from the health service following submission of dispensing records and a dispensing fee per item is also paid to pharmacists [3].

Expenditure on medicines under the GMS and community drug schemes increased greater than five-fold over the decade 1997 to 2007 [4], and in 2010 Ireland spent more on pharmaceuticals than any other European country on a per capita basis [5] with public expenditure amounting to €1.9 billion [6]. In the years 2011–2013, costs have remained at this level. Following this, the 2011–2016 Programme for Government in Ireland made commitments to reduce the State’s pharmaceutical bill and reduce costs for consumers, including through greater generic medicine usage [7]. Generic medicines provide an opportunity for savings on expenditure on medicines due to their typically lower price, and generic substitution policies aimed at promoting the utilisation of generic medicines have been introduced in several countries with considerable success [3–8]. In Europe, the rate of generic medicines use exceeds 50 % in some countries, and is rising slowly in other countries in tandem with a concerted cost containment policy for healthcare in the EU [9]. Underuse of generic medicines, meanwhile, is considered to be one of the leading causes of economic inefficiency in healthcare [2].

Ireland has traditionally had a low rate of generic medicines use with rates of approximately 18 % in the 1990s and minimal increases in the early part of the 21st century [10–12]. Postulated contributory factors for this low uptake include negative perceptions of generic medicines by prescribers and patients; relatively small price differential between proprietary medicines and their generic versions; the absence of any legal provision which either permitted or required pharmacists to undertake generic substitution; and the limited extent of prescribing using the drug’s international non-proprietary name (INN) [8]. More recently, generic uptake in Ireland was found to undergo a considerable increase during the period 2010–2012. In an analysis examining ten leading multiple-source off-patent pharmaceuticals, the market share of generic manufacturers for these products was found to increase from 24 to 50 % within the GMS scheme, and from 14 to 36 % within the DP scheme during these years [8].

Increases in generic medicine uptake are facilitated where reimbursement policies or financial incentives are firmly in place to promote the prescribing and dispensing of generic medicines [9]. The passing of legislation in Ireland, in the form of the Health (Pricing and Supply of Medical Goods) Act, 2013, has provided a mechanism for the introduction of generic substitution of medicinal products based on interchangeability at active substance level, and for a system of internal reference pricing [13]. Future enabling policy measures may include the introduction of compulsory INN prescribing, as recommended by the International Monetary Fund under the economic adjustment programme for Ireland [8]. The success of any measure to promote generic substitution depends, however, on the support of the key stakeholders, the barriers to substitution in terms of existing perceptions, and the supports in place to facilitate a smooth transition to generic substitution by pharmacists [9]. The aim of this study was to assess the knowledge and perceptions of patients, pharmacists and prescribers (specifically medical doctors) in early 2013 towards generic medicines and substitution, prior to the introduction and implementation of the Health (Pricing and Supply of Medical Goods) Act 2013 (July 2013).

## Methods

Quantitative survey methodology was used to achieve the study end-points. Surveys were developed for patients, prescribers and pharmacists following a literature review of published studies [14–19], and included both structured and unstructured questions. Surveys were piloted on a sample of all stakeholders prior to wider dissemination. Surveys contained 16 (prescriber survey), 20 (pharmacist survey) and 23 (patient survey) items in total. Patient surveys consisted of three sections: (1) characteristics of patient (age, gender, number of chronic diseases and number of prescribed medicines) (2) knowledge of a generic medicine, and (3) perspectives on previous generic substitution and willingness to accept substitution. The survey for prescribers and pharmacists consisted of two parts: (1) demographics of respondents (professional status, number of years in practice) and (2) assessment of their perceptions on generic substitution, acceptance level and perceived barriers. Ethical approval for the study was obtained through the St. James’s Hospital/Tallaght Hospital Research Ethics Committee (No. 2012/12/29).

Purposive sampling methods were used to identify respondents to facilitate data collection in the short duration of time available. Survey administration methods were stakeholder specific. One-to-one interviews were conducted with patients, all pharmacists completed an on-line version of the survey administered using Survey Monkey™, while prescribers either completed an on-line version or self-completed a paper version.

### Stakeholder recruitment

#### Patients

Patients were recruited in both the primary and secondary care settings. Patients attending community pharmacies where pharmacy students were training under the National Pharmacy Internship Programme were recruited over a 6 week period (June–July 2013). Patients attending an outpatient (OP) clinic in a University Teaching Hospital were recruited between April and May 2013.

All patients gave written consent prior to participation. Patients were interviewed face-to-face by pharmacy interns in community pharmacies and research nurses in the OP setting. Patients were aged 18 years or over. Children were excluded from the study, as were patients with known cognitive impairment.

#### Pharmacist and prescriber recruitment

Pharmacists registered with the Pharmaceutical Society of Ireland (PSI; the regulatory authority for pharmacists in Ireland) received an invitation to participate in the survey through the monthly electronic newsletter from the PSI with a link to the on-line survey URL. Surveys were completed between March 18th and April 1st 2013. Prescribers were invited to participate, either through a personal email from the study authors with a link to the on-line web URL, or by completion of a hand written survey distributed at educational fora for general practitioners in the University Teaching Hospital, between January and July 2013.

### Data collection and analysis

Patient and prescriber hand-completed surveys were entered into a coded database by one study investigator, while pharmacist survey data was downloaded from the on-line website (Survey Monkey™). Pooled datasets underwent a systematic quality control procedure to identify data anomalies. The patient dataset was screened for date of birth data anomalies, and implausible data entry errors. Anomalous data were checked with raw questionnaire forms and subsequently corrected. To validate the categorical variable data, a random sample of the patient questionnaires (10 %) were selected, and a second study investigator undertook a double data entry exercise. The datasets were matched and an error difference of <1 % was determined correlating with an acceptable difference. All prescriber questionnaires available for data entry underwent a double data entry process which again achieved a <1 % difference in rate of error. Narrative data received in response to open-ended questions in the three surveys underwent a process of enrichment where applicable, and answers were categorised based on identified themes. This was conducted by the first author who read and re-read the narratives to capture an overall sense. Themes and sub-themes were then established and checked by the second and third authors until consensus was reached.

Final datasets were imported into SAS (Version 9.1^®^) for descriptive and statistical analysis. Logistic regression was used to determine factors that predict a patient’s knowledge of generic medicine. Independent variables considered were age, gender and knowledge of generic medicines.

## Results

### Quantitative analysis

A total of 353 patients, 100 prescribers and 662 pharmacists (response rate 17 %) were included in the analyses. The rate of non-participation was not recorded for the patient or prescriber cohort due to the nature of sampling.

#### Patient cohort

In the patient cohort, 133 were recruited from a hospital-based out-patient department and 220 patients from primary care. There was a higher prevalence of female respondents over male (55 %), and 43 % of the total cohort were over 60 years of age. The mean number of chronic diseases per patient was 1.4 (range 0–7). Forty-two percent of patients qualified for free medicines under the GMS scheme, while 27 % paid for their medicines privately. Cardiovascular disease accounted for the co-morbid profile of 62 % of patients, while 37 % were prescribed more than 5 medicines.

#### Prescriber and pharmacist cohorts

Minimal demographic details were obtained for the healthcare provider cohort.

**Fig. 1:**
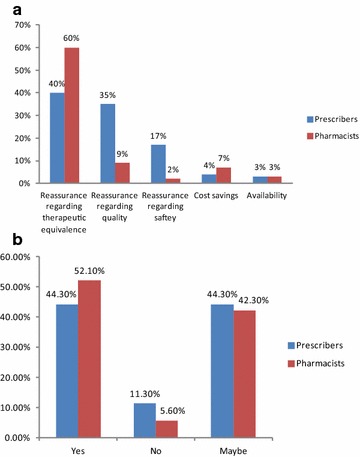
**a** Number of years in practice—prescribers. **b** Number of years in practice—pharmacists

The time in practice profile of respondents among prescribers was generally longer than that of the pharmacist cohort with 33 % practising for more than 30 years, as compared with the pharmacist cohort, where 40 % were in practice for less than 10 years (Table 1). Both cohorts were delivering healthcare almost exclusively in the primary care setting: 97 % for prescribers and 93 % for pharmacists (Fig. 1a, b).

**Table 1:** Area of practice for healthcare professional respondents

Area of practice—prescribers		Area of practice—pharmacists	
General practitioner	97 %	Community pharmacist	75 %
Hospital-based consultant	2 %	Hospital-based pharmacist	16 %
Non-clinical role	1 %	Non-clinical role	9 %

#### Patients’ knowledge of the term ‘generic medicine’

The majority of patients were familiar with the term ‘generic medicine’ (84 %) based on the accepted definition of the term. Familiarity with the term decreased in older patients with 64 % aware of the term in the >80 years age group. While the community-based patients were less likely to be receiving medication for cardiovascular disease [Odds Ratio (OR) 0.06, 95 % CI 0.03, 0.14], there was no difference between the groups with respect to their familiarity with/knowledge of the term “generic medicine” (OR 1.64, 95 % CI 0.88, 3.04).

Patients were asked a series of questions specifically examining their knowledge of the term ‘generic medicine’ (Table 2). Patients agreed that generic medicines were the same and cheaper than branded medicines (81 and 82 % respectively), while they were unsure whether generic versions were available for all medicines.

**Table 2:** Patients’ knowledge of generic medicines

	Yes (%)	No (%)	Do not know (%)
Generic medicines are the same as branded medicines	81	7	12
Generic medicines are as effective as branded medicines	76	11	13
Generic medicines are as safe as branded medicines	75	5	20
Generic medicines are available for all branded medicines	16	38	46
Generic medicines are cheaper than branded medicines	82	4	8

#### Patients’ acceptance of generic substitution by healthcare providers

When asked how willing they would be to accept a generic medicine for a branded product, 76 % of patients said they would be willing to do so if prescribed by their GP. This compares with 58 %, who would be willing to accept a generic substitute by the pharmacist.

#### Patients’ experience with generic medicines

Some 42 % of the cohort had a previous experience of switching from a branded to a generic medicine, with the majority of these initiated by the GP (73 %), although a smaller proportion reported switches initiated by the pharmacist (27 %). Of the 42 % of patients currently taking a generic medicine, a significant number reported a change in packaging (90 %) and a change in appearance or shape (67 %). However, despite these changes, it had little or no impact on self-reported compliance (Table 3).

**Table 3:** Patients’ experience with switching to a generic medicine

	Yes (%)	No (%)
Change in packaging	90	10
Change in shape or appearance	67	33
Effect on compliance	14	86

#### Prescribers and pharmacists support for generic medicines and generic substitution

Prescribers supported generic substitution for original branded drugs in most cases (74 %), and agreed there were some situations where it was not appropriate. This compares to 84 % of pharmacists who supported generic substitution in most cases. Of the prescribers surveyed, 20 % supported generic substitution for original branded drugs in all cases where a generic was available, compared to 16 % of pharmacists. Overall, support for generic substitution was evident in the responses provided.

Both prescribers and pharmacists were broadly in agreement with the types of information they considered important to impart to patients with therapeutic equivalence and quality of generic medicines considered of greatest importance by both healthcare professionals (Table 4). Both stakeholder groups agreed that patients were accepting of the concept of generic substitution, with 44 % of prescribers responding in favour, compared to 52 % among pharmacists. Both groups considered it likely that a further 40 % or more of patients could be willing to accept generic substitution. A comparison of other perspectives of healthcare professionals on generic substitution is shown in Table 5. The influence of hospital-initiated prescribing is indicated by the proportion of healthcare professionals who would be less likely to undertake substitution if a branded product was prescribed in the hospital setting (13–16 %).

**Table 4:** Perceptions of healthcare providers around importance of information to be provided to patients

	Prescribers (%)	Pharmacists (%)
Therapeutic equivalence	40	60
Quality	35	19
Safety	17	12
Cost savings	4	7
Availability	3	3

**Table 5:** Comparative views of healthcare providers (prescribers and pharmacists) on generic substitution

	Yes (%)	No (%)	Maybe (%)
Do you think patient care may be affected in any way by generic substitution?			
Prescriber	24	51	25
Pharmacist	14	48	38
Would you be more likely to opt out of prescribing/dispensing a generic medicine is a hospital prescriber had prescribed a branded product?			
Prescriber	16	62	21
Pharmacist	13	68	20
Are you comfortable with the concept of generic substitution of a branded medicine?			
Prescriber			
If undertaken by a pharmacist, of a branded product prescribed by you	58	18	23
Pharmacist			
With undertaking generic substitution of a branded product prescribed by a prescriber	86	3	12

### Thematic analysis of narrative comments

#### Patients’ views on generic medicines

Narrative comments which were suggestive of potential patient barriers to generic substitution included references to lack of comparable effectiveness to branded medicines (often grounded in experience), absence of confidence in generic medicines and experience of adverse effects.

“….not as good as….don’t work as well…”

Patients described the potential for confusion and anxiety with changes in packaging and appearance.

“…..Confusing as different names on boxes… stickers cover the information…”.

A number of patients felt that there was a need for more research into the effectiveness of generic medicines.

“…..Felt better on branded medicine, felt it worked better…”

A number of patients stated a preference to stay on branded medicines rather than having to accept a generic medicine. The need for patients to be given additional information and time to consider generic substitution was fed back in open ended comments.

Several patients provided positive comments on generic medicines, and stated they were happy on generic medicines, and had no problems with them or experienced no adverse sequelae. Some went further to state that it made them more alert when switched.

“….It made me more alert and improved my compliance…..”

#### Prescribers and pharmacists views on generic medicines and substitution

Prescribers were particularly concerned about the effect of generic substitution in vulnerable patient groups including the frail, the elderly, those on multiple medicines, those with cognitive impairment and patients suffering from anxiety. This was similarly represented in pharmacist feedback. A significant number of healthcare professional respondents commented on the potential for generic medicines to cause adverse effects, and for a consequent adverse impact on patient compliance, ultimately leading to therapy failure. The potential effect of such drug-related problems on patient welfare was thought to be particularly worrying for patients with long-term chronic conditions including epilepsy and mental health problems. A contributory factor suggested by prescribers was possible confusion arising from repeated changes in packaging should different generic medicines be dispensed to patients each month depending on cost.

“….There must be provisions made that prevent the changing of a patient’s generic medication every other month i.e. just because A is 1 cent cheaper than B another substitution should not be made. This would be extremely confusing for patients and health care……”

The availability of ‘branded generics’ in Ireland was also postulated to contribute to potential confusion among patients.

…..It would be better if generic products didn’t have their own brand names….

For both pharmacists and prescribers, there was consensus on the need for specific drugs to be exempt from substitution for clinical and safety reasons. This was especially true for drugs with narrow therapeutic indices such as antiepileptic drugs, modified release formulations, multiple ingredient products and the unusual situation when different brands were licensed for different indications. Both cohorts were consistent in the explicit and comprehensive examples of medicines considered appropriate to exempt.

There were additional, specific concerns highlighted in relation to the absence of firm evidence regarding the therapeutic equivalence and the quality of generic medicines.

…….‘Not convinced of equal efficacy of generics- more evidence needed’….

The potential for altered formulations and additional excipients to cause adverse effects was raised.

Both stakeholders alluded to the limited potential for actual cost savings to be made from generic substitution in Ireland given the small price differential between generics and branded medicines in place in Ireland in the past.

….‘Biggest problem is the high cost of generics, with little or often no savings available’……

A practical barrier to effective implementation of generic substitution identified in the responses was computer software systems in GP practices, which do not facilitate a ‘*user friendly system to allow generic prescribing/substitution by GP computer system’.*

In general however there was a positive attitude towards generic medicines and generic substitution.

….‘Generic prescribing is long overdue, I have prescribed generically for years. My patients gained significantly with no loss of effectiveness’…..

## Discussion

The aim of this study was to assess the knowledge and perceptions of Irish patients and Irish healthcare providers to generic medicines and generic substitution prior to the implementation of the Irish nationwide generic substitution policy. Our findings suggest that the majority of patients demonstrated a good understanding of generic medicines (84 %). Another recent study of patient perceptions of generic medicines in the Irish setting, reported that 31 % of patients had no knowledge of generic medicines, compared to the lower rate of 16 % in our study [20]. This may be explained by the different method of surveying patients, i.e. in-depth qualitative interview technique as compared with survey methodology. The potential for acquiescence bias may be higher with survey methodology than face-to-face interviewing. Over 80 % of our patient cohort was aware that generic medicines are the same as branded medicines and less expensive, while 75 % perceive them to be as effective and as safe as their branded counterparts. Although previous studies have reported that patients perceived generic medicines to be inferior or not as effective as branded products (mainly due to the price differential between the two implying generics were of lower quality) [14, 18, 21–23], this perception did not dominate the feedback in our patient cohort. Patients were, however, less sure of the availability of generic medicines for all branded products, perhaps not a surprising finding. This overall enhanced knowledge among patients of generic medicines may in part be due to widespread publicity across the media in the lead up to the passing of the Health (Pricing and Supply of Medical Goods) Act in July 2013 which introduced generic substitution. This would appear to support the benefits of providing education and information campaigns to inform the public. This is underpinned by the recent review by Hasali et al. who reported an upward trend in patient knowledge and confidence towards the use of generic medicines following introduction of generic substitution policies, and a similar trend may become apparent here [24].

Patients also reported a high rate of willingness to accept substitution from either prescribers or pharmacists, although there was an indication that GP-initiated substitution was favoured over pharmacist-initiated substitution. Further education initiatives would be useful in providing further reassurance to patients who have remaining doubts about the effectiveness of generic medicines, or may be resistant to change, as has been reported previously [14, 18, 21, 22, 25]. Appropriate and adequate patient education for patients prior to switching is necessary, and there is robust evidence that an interdisciplinary approach can optimise patient acceptance to generic medicines and substitution. Healthcare professionals play a significant role in educating patients about generic medicines, and several studies have highlighted positive endorsement from prescribers and pharmacists as important drivers for patients accepting a generic substitute [14, 26–29]. The input of both prescribers and pharmacists is therefore required to improve confidence of patients in generic medicines, and to accept generic substitutes for branded medicines. This may incur additional time explaining the concepts and rationale for generic substitution to their patients, which in the time constrained primary care setting may prove difficult.

Almost half of those patients surveyed had experienced a switch to a generic medicine in the past. This was associated with significant changes in packaging, shape and appearance compared to their existing medicines, and this could occur repeatedly on subsequent occasions when the medicine was dispensed. Previous studies have reported on the potential for generic substitution to result in significant patient confusion, and anxiety [17, 22, 30]. While confusion was reported among some patients who were switched in this study, it was not reported to impact on compliance. It is acknowledged however, that the reliability of self-reported compliance as captured in this survey method may be associated with considerable uncertainty [31]. A recent US study has reported increased odds of non-persistence associated with changes in pill colour among patients with epilepsy [32]. While persistence is a surrogate marker of compliance, the need to minimise frequent changes in packaging should be addressed through consultation with pharmacists, who have a professional responsibility to ensure that changes do not impact negatively on patients through appropriate patient counselling, focussed stock management and improved terms from suppliers of generic medicines. Restriction on the availability of branded generics may also be merited to avoid confusion.

The majority of healthcare professionals supported generic substitution in ‘most cases’, with a larger proportion of pharmacists supportive (84 %) compared with prescribers (74 %). The new Act implemented in 2013 provides that all pharmacists in Ireland will now be required to offer patients the opportunity to substitute a prescribed non-generic, interchangeable medicinal product with a less expensive generic alternative. Previous to the Act, pharmacists were required to dispense the medicinal product as prescribed by the healthcare provider. Where the patient declines the substitution of a medicinal product, the price of which is at or below the reference price set for that product, the patient will be responsible for paying the pharmacist the difference between the reference price and the price of the branded product dispensed. There is provision in the Act whereby a prescriber of a non-generic medicine may specify on the prescription “do not substitute” beside the name of the medicinal product concerned. The apparent rationale for this provision is to acknowledge a prescriber’s professional discretion in this regard which they would normally exercise to protect patients with specific clinical needs from being adversely impacted by substitution. Similarly, there is a provision for patients to opt out of accepting generic medicines, but will be obliged to incur the additional cost of the difference in price between the generic medicine and the branded medicine. There are no financial incentives associated with implementation of the Act.

There was a correspondingly high level of ‘comfort’ with the concept of a national generic substitution policy, which is encouraging. Both prescribers and pharmacists perceived that patients would be willing to accept generic medicines, which was supported by patients’ perceptions as reported in this study. This may have stemmed from previous experiences of healthcare professionals with switching patients, and provides evidence of a baseline positive attitude to the pending national policy, which should impact in a constructive way on rates of uptake for generic substitution.

Misconceptions among healthcare professionals about generic medicines and generic substitution have previously been reported in the literature as barriers to effective implementation of generic substitution policies [14, 15, 33]. While most healthcare professionals captured in this study did not perceive that substitution of branded medicines with generic versions would impact negatively on the overall clinical care or therapeutic control of patients, concerns were raised in relation to specific patients particularly in frail, elderly, cognitively impaired patients and those on multiple medicines. This is similar to previously published reports of attitudes of prescribers and pharmacists to generic medicines [17, 21, 30, 33, 34]. The potential for negative consequences on compliance if several medicines were switched simultaneously, sequentially or repeatedly was reported in this study. A small number of studies have reported decreased adherence associated with generic substitution [33, 35, 36], although whether there may be an effect on patient compliance in the Irish setting is unknown as yet, and may prompt the need for prospective persistence studies to assess the impact, if any. From a patient perspective, as self-reported by patients surveyed, compliance was unaffected despite experiencing changes in packaging. This confirms the absence of an effect on compliance as previously reported by Van Wijk and Oleson [37, 38]. However, the degree to which social desirability may have confounded responses among patients in this study is unknown, but is recognised in the literature [31, 39].

Low perceived efficacy and safety is reported by the WHO, as a common reason for generic medicines’ underuse, in addition to prescribers’ concerns in relation to efficacy and therapeutic equivalence [40–42]. Concerns have also arisen where small differences in bioavailability have led to questions regarding true bioavailability, particularly in relation to drugs with narrow therapeutic indices [43]. Prescribers in particular, in our study, had reservations around the bioequivalence of generic medicines. This perception was previously reported among Irish prescribers in the 1990s and also recently, so there appears to be a sustained uncertainty around generic medicines [10, 19]. Despite comments to the potentially inferior quality of generic medicines as compared with branded products, there is little in the literature to substantiate these claims.

Both prescribers and pharmacists provided several examples of specific medicines that they considered inappropriate for substitution, similar to those reported in the published literature [19, 44, 45]. These primarily focused on drugs with narrow therapeutic indices i.e. antiepileptic drugs, digoxin, thyroxine, warfarin, in addition to multi-constituent and sustained-released formulations. There is no provision in the legislation for particular exempted medicines, but there is provision for prescribers to specifically state on prescriptions that the medication is not to be generically substituted. As provided for in the Act, however, one of the factors which render a product unsuitable to be added to the interchangeable list is if it has a narrow therapeutic index, which would mean it could not be safely substituted.

The supply and pricing arrangements for generic products have also been reported as possible deterrents to effective generic substitution implementation. While cost savings have been reported in other jurisdictions [21, 46], it has been reported that such policies may increase costs due to the impact on patient compliance and adverse clinical effects [47] and therefore erode any potential cost savings in the long term [45]. Doubts were raised in this study by healthcare professionals as to the actual cost savings that could be achieved following the introduction of the generic substitution policy. This was attributed to the relatively minor price differential that existed between branded medicines and their generic equivalents in the past, together with the availability of so-called “branded generics”. It will be important to monitor generic medicine use following the introduction of the new generics policy and to analyse and publicise actual cost savings achieved obtained from its implementation to all key stakeholders on an on-going basis, to demonstrate its benefits.

Patients, prescribers and pharmacists alluded to the potential increased risk of actual adverse effects associated with generic medicines over branded products. Some of this was postulated in feedback to relate to altered excipients and manufacturing ingredients. Excipients or inactive ingredients in generic medicines may differ from originator medicines [48]. Adverse events or allergies to such ingredients have been reported in the literature, but the extent of the problem is low [48]. However, vigilance regarding the potential for harm is recommended among healthcare professionals, with some jurisdictions going so far as to advocate increased surveillance and reporting systems focused on adverse event reporting for generic medicines [49, 50].

Both GPs and pharmacists referred to the need for improvements to GP computer software systems to facilitate easier selection when issuing prescriptions of generic medicines from the ‘picking list’, or the most cost effective generic medicine. This might support the introduction of a more user friendly information technology system that facilitates selection of generic medicines for prescribers, within the confines of a limited fixed time consultation, or the availability of a national formulary medicines’ list.

### Strengths and limitations of the study

While this study investigated simultaneously the perceptions of the key stakeholders in Ireland to generic medicines, it has some limitations. The purposive sampling method used for recruitment may not reflect the broader views of these stakeholders, and therefore generalisability to the entire population of stakeholders may be limited. In particular, the views of prescribers may be underrepresented in this study due to the small sample size. In addition, the prescriber cohort was overrepresented by physicians with many years in practice whose responses may be subject to bias. The large sample size obtained for the pharmacist survey is less open to external validity bias. The patient cohort may also be less open to selection bias as the sample was drawn from a broad spectrum of patients who obtain their medicines through a variety of subsidised schemes. However, the extent to which the sample size limits generalisability to the wider patient population is unknown.

An additional limitation of the study centres on the reliability of self-reported compliance. The patient cohort who had previously experienced generic substitution reported that this experience had not impacted on their compliance. However, the reliability of self-reported compliance is weak due to a number of contributory factors which leads to the potential to overestimate adherence. These factors include recall bias, social desirability bias and errors in self-observation [31]. The authors also acknowledge the potential for researcher bias in the conduct of the patient interview due to the large numbers of individual interviewers in the study. Finally, while pilot testing of the survey was undertaken, the potential for the wording of questions to give rise to bias is associated with uncertainty.

However, a useful insight into the views of stakeholders has been obtained in the period immediately preceding the introduction of legislation permitting generic substitution.

## Conclusion

The findings from this study identify the high rate of acceptance of patients, prescribers and pharmacists in the Irish setting to generic medicines and participation in generic substitution. The existing concerns of the main stakeholders centrally involved in the process of generic substitution provide a valuable insight into the potential difficulties that may arise in its implementation, and the need for on-going reassurance and proactive dissemination of the impact of the generic substitution policy. There is a clear need to ensure that healthcare professionals are appropriately supported to ensure the additional time commitment involved in reassuring patients is minimised. The need for ex-post assessment of the legislation’s impact is apparent, and should be provided for. The existing positive attitude to generic medicines and generic substitution among the key stakeholders, combined with appropriate support and collaboration, should result in the desired increase in rates of prescribing, dispensing and use of generic medicines, with an associated saving in the annual expenditure on medicines in the Irish setting.

## Abbreviations

GMS: General Medical Services
LTI: Long Term Illness
DP: Drugs Payment
HTD: High Tech Drug
EU: Europe
INN: International non-proprietary name
OP: outpatient
GP: general practitioner
